# FIGENIX: Intelligent automation of genomic annotation: expertise integration in a new software platform

**DOI:** 10.1186/1471-2105-6-198

**Published:** 2005-08-05

**Authors:** Philippe Gouret, Vérane Vitiello, Nathalie Balandraud, André Gilles, Pierre Pontarotti, Etienne GJ Danchin

**Affiliations:** 1 Phylogenomics Laboratory. EA 3781 EGEE (Evolution, Genome, Environment), Université de Provence, Case 36, Pl. V. Hugo, 13331 Marseille Cedex 03. France; 2 AFMB-UMR 6098- CNRS - U1 - U2 Glycogenomics and Biomedical Structural Biology Case 932, 163 Avenue de Luminy 13288 Marseille cedex 09, France

## Abstract

**Background:**

Two of the main objectives of the genomic and post-genomic era are to structurally and functionally annotate genomes which consists of detecting genes' position and structure, and inferring their function (as well as of other features of genomes). Structural and functional annotation both require the complex chaining of numerous different software, algorithms and methods under the supervision of a biologist. The automation of these pipelines is necessary to manage huge amounts of data released by sequencing projects. Several pipelines already automate some of these complex chaining but still necessitate an important contribution of biologists for supervising and controlling the results at various steps.

**Results:**

Here we propose an innovative automated platform, FIGENIX, which includes an expert system capable to substitute to human expertise at several key steps. FIGENIX currently automates complex pipelines of structural and functional annotation under the supervision of the expert system (which allows for example to make key decisions, check intermediate results or refine the dataset). The quality of the results produced by FIGENIX is comparable to those obtained by expert biologists with a drastic gain in terms of time costs and avoidance of errors due to the human manipulation of data.

**Conclusion:**

The core engine and expert system of the FIGENIX platform currently handle complex annotation processes of broad interest for the genomic community. They could be easily adapted to new, or more specialized pipelines, such as for example the annotation of miRNAs, the classification of complex multigenic families, annotation of regulatory elements and other genomic features of interest.

## Background

Detecting genes, their organization, structure and function is a major challenge of the genomic and post-genomic era. Two fields of genomic biology are dedicated to this task and are known as structural and functional annotation. Structural annotation refers to the task of detecting genes, their location on a biological sequence, their exon/intron structure and predicting the protein sequences that they encode. Functional annotation aims to predict the biological function of genes and proteins.

Structural annotation methods can be classified into several types:

• Ab-initio methods, based on content sensor and detectors to discriminate between coding and non-coding regions, and then decipher a putative gene.

• Homology-based methods use evolutionary conservation concepts to deduce gene localization and structure.

• Hybrid methods couple these two approaches and usually present the best compromise in terms of sensibility and specificity in gene detection [1].

Computational methods of functional annotation are mainly divided into two types:

• Similarity based approaches intending to infer a function based on the pairwise similarity of a given sequence with a sequence of known function. These approaches have been criticized for their propensity of propagating annotation errors [2] deducing false homology relationships [3,4], and thus producing systematic errors [5].

• Phylogenomic inference approaches, based on evolutionary history and relationships between biological sequences. These methods avoid most of the false homology inference problems, and allow distinguishing between orthologous and paralogous genes [4,6]. Orthologous genes, which are produced by a speciation event, are more likely to share the same function than paralogous genes which originate from duplications [7]. These methods are also able to detect potential functional shifts through the study of genes' evolutionary behavior, [6]. Nevertheless, these methods require a high degree of biological expertise, are time consuming, complex, and are difficult to automate in their whole [4,8,9].

Aside from detecting protein coding genes and predicting their function, structural and functional annotation also have other aims such as detecting regulatory elements, repetitive elements, non protein-coding genes (i.e. miRNA), or other important genomic features.

Whatever the objective, structural and functional annotation usually require the complex chaining of various different algorithms, software and methods each with its own particular set of parameters and output format. At key steps of these "pipelines", expert biologists are often required to make important decisions, modify the dataset, compare intermediate results, manually handle and convert several files (and so on...) which is labor intensive and can be error prone. For the treatment of huge amounts of data released by sequencing projects, automation of these pipelines is an absolute necessity. Several attempts have been made in the development of annotation platforms automating some of these pipelines, particularly in the field of structural annotation (for example the Ensembl pipeline [10], or the Otto system [11]). With regards to functional annotation, several platforms automate pairwise similarity based approaches [9,10,12,13], and fewer have automated the more complex phylogenomic inference approaches [4,14]. While these latter platforms allowed both a gain in the time cost and avoid errors due to the manual manipulation of files, they still strongly require intervention of human experts at various steps.

Here we present an automated annotation platform featuring an expert system that substitutes for human expertise at various steps and, thus, allows more complete automation than ever considered. The expert system models the biologists' expertise and is able to compare intermediate results from different methods, to modify the dataset, to evaluate the significance of predictions along with other usually "biologist-made" tasks. The FIGENIX platform currently automates 8 different pipelines of structural and functional annotation. In particular, a structural annotation pipeline, which is a hybrid method coupling ab-initio and homology-based approaches, and a functional annotation pipeline fully automating a complex phylogenomic inference method. The present manuscript will specifically focus on the phylogenomic functional inference pipeline which illustrates how an expert system allows automation of complex chaining usually requiring amounts of non-trivial human intervention.

## Implementation

FIGENIX is an intranet/extranet server system usable through any recent Web browser accepting JAVA 2 Plugin installation. FIGENIX is freely available to academic users through the web interface [15]. Users first have to contact us to request a login and password. The source code is available upon request under the GNU GPL (General Public License).

### FIGENIX's technical architecture

**Figure 1:**
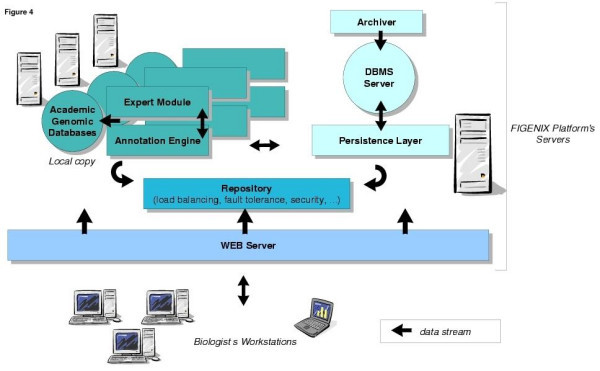
**FIGENIX software architecture. **FIGENIX software servers can be distributed on several CPU. Some servers, like "Annotation Engine" or "Expert System" can be cloned and distributed on these CPU (theEnsembl pipeline includes a similar approach, named "Computer Farm"). This allows load balancing inside the FIGENIX platform. Fault tolerance is not yet implemented but can easily be integrated to this kind of architecture.

The FIGENIX (1.0) platform is structured as a 3 tiered software which means that it is composed of three layers: the database management system, the server-side components and the graphical user interfaces (Figure 1). Software components are distributed through JAVA RMI middleware technology, on our laboratory's network. To increase computation capabilities and to further offer FIGENIX services to the biologists' community, deployment on GRID middleware architectures like UNICORE is technically possible and can be considered.

For software development and production deployment, we chose the LINUX operating system (for production we use RED HAT 9.0 open access version), for several reasons: UNIX kernel reliability, free and open source software and especially the availability of algorithmic software widely used for genomic annotation in their command line version (e.g. BLAST [16], GENSCAN [17], HMMGENE [18], CLUSTALW [19], PAUP* [20], PHYLIP [21], TREE-PUZZLE [22], HMMPFAM [23]). The Relational Database Management System, which is responsible for the persistence of annotation tasks (pipelines instances) and the genomic results produced during tasks executions, is POSTGRESQL [24]. Server-side components (see figure 1) are developed in the JAVA language [25] (exhaustive list of used technologies: JAVA, RMI, SWING, JSP, TOMCAT, HTML, JAVASCRIPT, XML, XSLT, POSTGRESQL, GNU PROLOG FOR JAVA, BIOJAVA, FORESTER, C). A server called "Persistence Layer" manages "Objects" mapping for tasks and their results in the relational database server. A "Repository" centralizes information concerning operating conditions of FIGENIX (e.g. the load balancing ticket between "Annotation Engines"). The "Annotation Engine" is a component able to execute several annotation tasks at the same time (multi-threading) and, thus, to drive several pipelines. The Engine works with local copies of genomic databases, automatically downloaded and updated from academic Web sites (NR, SWISSPROT/NCBI [26], Ensembl [10], PFAM [27]). We do not include a task manager like the "Rule Manager" of the Ensembl pipeline [12], but rather all tasks to be run are placed in the database server and each "Annotation Engine" looks periodically if some tasks can be executed on its CPU. The "Annotation Engine" obligatorily works in conjunction with an "Expert System". This module integrates static empiric rules associated with genomic knowledge extracted from the laboratory scientists and dynamic information given during the pipeline execution. The static knowledge base is shared by all tasks executing on the engine asking expert, but each task owns its "world" which encapsulates its specific dynamic information (e.g. multiple alignments, domains, temporary phylogenetic trees that are given to the expert system or tests' results).

This module was developed with PROLOG language (see Table 1) (Colmerauer, unpublished, 1972) with GNU PROLOG FOR JAVA interpreter [28]. Based on first order logic, it offers easy knowledge modeling using logical rules. This language is very well adapted to data structures like lists and trees. In bioinformatics solutions, these kinds of structure are numerous and common. In a natural way, a PROLOG engine (interpreter) works in "backward chaining" mode, i.e. like a predicate verifier and not like a facts producer ("forward chaining"). In other words, it answers to questions rather than producing new information. This mode is well appropriated to the way the "expert system" takes part in task execution (as illustrated in the examples detailed hereafter). As shown further, the choice of a rule based system like PROLOG, offers a great "expression capability" in very short and powerful sentences used to model scientists' knowledge and methods. Procedural or "Object Oriented" languages (like C/C++/C#, JAVA, PERL, or PASCAL) do not offer such powerful, concise and interpreted syntax for knowledge modeling and manipulation purpose.

**Table 1:** PROLOG rules, syntax and semantic, example

*% X belongs to a list if X is at the start of the list*	
***Element(X, [X|_])*.**	
*% or X belongs to a list if the list starts with Y different from X but X belongs to the list 's queue*	
***Element(X, [Y|;L]) :- different(X, Y), element(X, L)*.**	
	
*a user of a such program asks it like this:*	**Answer**
***>element(9, [5, 3, 4, 7]*.**	*answer = fail*
***>element(3, [5, 3, 4, 7]*.**	*answer = ok*
***>element(X, [5, 3, 4, 7]*.**	*answer = ok, X = 5 or X = 3 or X = 4 or X = 7*

a(X, Y) :- b(X), c, d(X, Y) can be read like this: a is true for X and Y values if b is true for X value, c is true and d is true for X and Y value. Here is a very short example to explain how works a PROLOG program. Suppose we want to write a program able to verify that an element belongs to a list or able to enumerate list's elements. In PROLOG we just have to describe "belonging" concepts.

### Complex pipeline example: phylogenomic inference

As an example to illustrate the potential of an expert system in automating complex and human intervention-requiring pipelines; we focused on the phylogenomic functional inference pipeline. Phylogenomic functional inference is, as previously introduced, labor intensive, time consuming, requires a high level of expertise and human intervention at various different steps. For these reasons, such functional annotation approaches, while clearly more reliable than similarity based approaches, have been considered as impossible or very difficult to automate without dramatically sacrificing the quality (by substituting general default parameters and decisions to human expertise).

**Figure 2:**
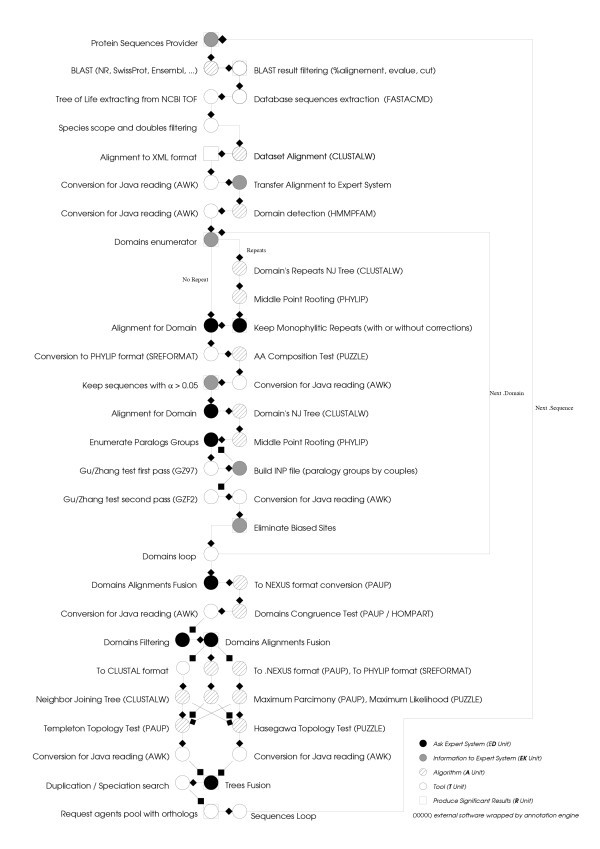
**Phylogenomic inference pipeline. **For more details about all the steps and functionalities automated in the pipeline see material and methods sections of the 2002 and 2003 phylogenomic papers [29, 30]. From the query sequence, a dataset of putative homologous sequences is first built by BLAST [16] on a protein database like NR. We filter raw dataset to eliminate sequences potentially non-homologous, disturbing alignments and doubles. User can choose to focus on a specific scope on any node of the tree of life (the vertebrates, the bilaterians...). In the next step, we produce an alignment with CLUSTALW [19]. Then the alignment is modified to eliminate large gaps. Since phylogenetic analysis is done at the domain level, we next detect these domains with HMMPFAM [23]. For each domain alignment (extracted from the original alignment), a bias correction phase is run, to eliminate: – Non-monophyletic "repeats" in a tree built with NJ [31] algorithm on CLUSTALW software. – Sequences with a diverging composition by using an amino-acid composition test of TREE-PUZZLE software [22] (with an alpha risk set to 5%). – Sites not under neutral evolution [35]. Once domains are "purified", and after congruent domains selection with HOMPART test from PAUP package [20], a new alignment is built by merging preserved parts of domains' alignments. From this alignment, three phylogenetic trees are generated using NJ, ML (with TREE-PUZZLE [22]) and MP (with PAUP [20] package) methods. By comparing topologies of these trees with PSCORE command ("Templeton winning sites" test) from PAUP package and KISHINO-HASEGAWA [34] test from TREE-PUZZLE package, fusion of these trees in a unique consensus tree is produced. Through the comparison of this consensus protein tree with a reference species tree, (the tree of life from NCBI [26]), we then deduce orthologous proteins to the query sequence.

The phylogenomic inference pipeline that we integrated and automated in the platform is mainly the one described in Abi-rached et al. 2002 [29], and in Vienne et al. 2003 [30], the pipeline is described on Figure 2's legend.

Phylogenomic inference can be summarized into five main steps:

1. Creation of a dataset of sequences homologous to the sequence of interest.

2. Multiple alignment of these sequences, with elimination of data producing bias, noise, or distorting the evolutionary signal.

3. Phylogenetic reconstruction based on the multiple alignment using several different methods.

4. Inference of Orthologs and paralogs through comparison of gene trees with a reference species tree.

5. Retrieval of experimentally verified functional data for orthologs and paralogs to the query sequence, on Web databases (Gene Ontology, MGI and NCBI's dbEST).

In each of these five steps, human intervention is required multiple times. For example, at step 1 to choose sequences from a BLAST [16] output that are more likely to be homologous to the query sequence. At step 2 to eliminate sequences producing biases in the alignment or having a divergent composition, and to mask sites with highly divergent evolution. At step 3 to compare the topologies of trees produced by different methods and check whether they are congruent. At step 4 the biologist compares the topology of the gene tree to the topology of a reference species' tree, then deduces the position of duplication and speciation events, and finally infers orthology and paralogy relationships. Once orthologs to a sequence of interest have been identified, biologists then usually look for known functional data in other species and infer for example a likely biochemical function for the unknown gene (step 5).

### A complex pipeline's computational translation

**Figure 3:**
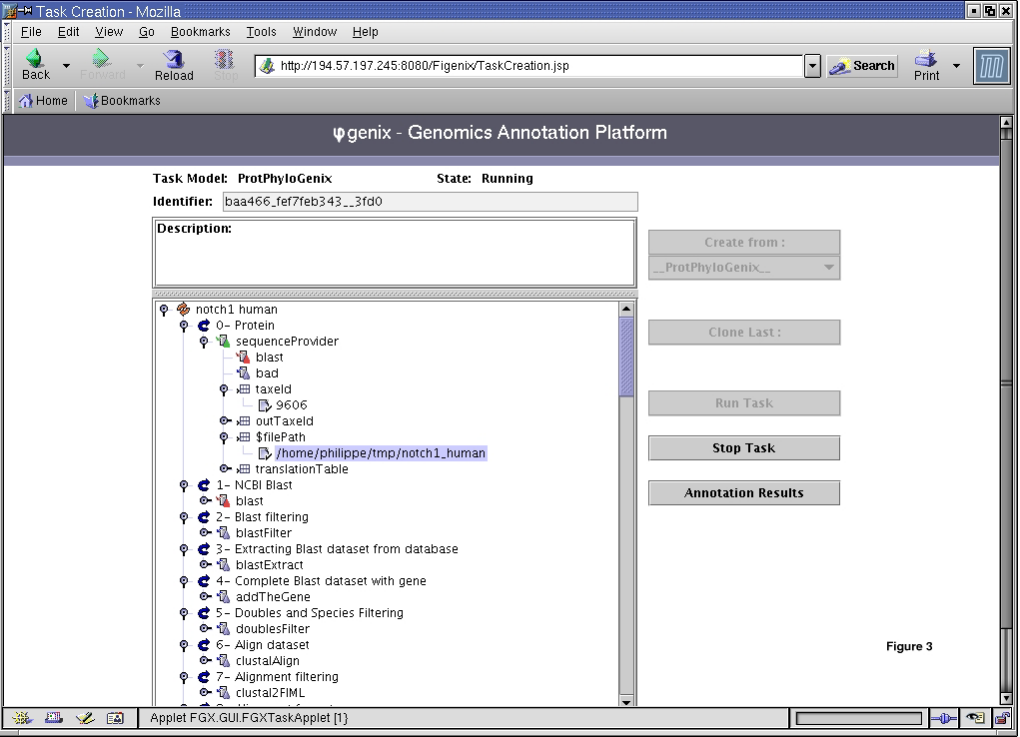
**Task creation and running (GUI). **Here is shown a phylogenomic inference task on human Notch1 protein. The graph associated to the phylogenomic pipeline is displayed on the left part of the figure, as a graphical tree. We introduced a virtual concept of "step of work" that allows to show a cyclic oriented graph as a tree. At each step one or several units can act. (e.g.: at the step named "Protein", the unit "sequenceProvider", whose role is to read protein sequences from a file, will work). At the level just next to the current unit, are represented the units that will be activated as its continuation. (e.g. "BLAST" unit follows "sequenceProvider" cause the first treatment executed on a protein is the BLAST search). At the same graphical level as nodes related to a unit, are shown the parameters which can be customized for this unit (e.g. on "sequenceProvider" unit, the parameter "taxeid" (the query sequence's taxon) or parameter "$filePath" (path to the file with proteins to be analyzed)). The task given as an example in the figure was currently running when we took the screenshot. In green are shown units that finished their work, in red those which are running, in blue those which are not running. One can guess, by observing buttons on the right part of the figure, that the presented task: is an instance of pipeline model named "__ProtPhyloGenix__" (the one which produces phylogenomic inference studies for proteins), can be interrupted at any time, can be cloned (when user want to run it again modifying only several parameters), and finally explored through the scientific results web pages already produced according to the execution.

From a data-processing point of view, a procedure, such as phylogenomic inference introduced here, consists of a genomic data flow circulating through a software unit set. This data flow or "pipeline" is a directed cyclic graph (see figure 2 and figure 3).

Each graph's node, i.e. each unit takes one or more streams as an input and builds a new stream as an output, which is transferred to the input of one or many related units in the stream orientation.

Unit's jobs can be executed in a parallel mode. A "rendez-vous" type, synchronization, which means that a unit starts its work when the complete set of related input streams are present, is thus possible (see the 3 phylogenetic trees building units on figure 2) but not mandatory (unit's work can be started by the arrival of a unique input stream). This kind of parallelism, with explicit and large granularity, at the unit's level, allows us to benefit from multi-processors hardware architecture, and also, by an appropriate deployment, from distribution on several CPUs.

We name algorithmic or "A-units", units that produce a mathematical computation.

Like other adaptable and flexible pipelines systems, we didn't choose to rewrite new software for each algorithmic step. We preferred the use of the "reference" publicly available software in their command line version (e.g. sequence similarity search is done by the BLASTALL local runtime, downloadable on NCBI web site). Thus the BLAST process is driven by a "A-unit" which wraps its input/output streams.

We used the same approach for all software (gene prediction, domain detection, phylogenetic reconstruction, multiple alignment...). Plugging of existing software without modification in our pipelines, allows us to use the most advanced bioinformatics software research development, with a very easy maintenance. It also allows easy evolution of the platform by integrating new software or replacing the older versions by the most up to date ones. New versions of applications (such as BLAST, or HMMPFAM) are not directly and automatically updated in FIGENIX, they are first tested, validated and if needed adapted (due to possible changes in the input/output formats).

The "tool" units, or "T-units" category contains units like enumerators, data accumulators, multiplexers/demultiplexers, simple filters, data converters and so on (e.g. converting data from GENSCAN output data to GFF format).

"Result" units, or R-units, are in charge of the most important genomic results production. Those results are intended to be the components of a scientific report produced by an annotation task started by the biologist.

Interface with the expert system is made through two types of dedicated units. Their role is to "substitute to" human expertise and "memory". Some of them keep information necessary for later reasoning, they are named expert knowledge units, or EK-units. Others take decisions concerning stream direction inside the graph or produce, on output stream, new data resulting from the analysis of the current situation in the data world of the task. These units are named expert decision units, or ED-units. This part of the analysis is based on empiric rules specified by biologists, rather than on an algorithmic approach.

EK and ED units are thus gateways to the expert system, which purpose is to take decisions, using genomic knowledge and data provided by EK units during pipeline processing. Like a human, this expert system has a "memory" and an "intelligence" (limited to the problems managed by our system) used to "supervise" a pipeline execution.

Pipelines themselves are coded as XML files. We are developing a GUI (graphical user interface) for pipeline editing, dedicated to the biologists' use. Scientists will be able to construct their own data flows, chaining available tool units. A semantic control will prevent invalid buildings. Users can propose a given application not currently available in FIGENIX to be included as a new A-unit. This allows for example to substitute a new more accurate or more adapted application to the application currently used in the available pipeline. Users can also decide to share their custom pipelines with other FIGENIX users.

### Expert system usefulness examples

To illustrate the importance of a rules-based system, we selected two key examples in which the expert system substitutes for human expertise to take important decisions, to compare intermediate results, or deduce biological information.

One simple example is from step 3 of the phylogenomic inference approach summarized previously, which consists of reconstructing phylogenetic trees from a multiple alignment, then comparing the topologies of trees produced by different methods and producing a unique consensus tree on which all data are projected. The other more complex example is from the step 2, which consists of producing a reliable multiple alignment with elimination of sequences and masking of positions producing biases in the alignment or improper for phylogenetic reconstruction. This step is crucial in the phylogenomic approach because depending of the quality of the alignment in terms of phylogenetic signal and noise, a reliable phylogeny may not be able to be produced.

#### Example 1: trees consensus

In FIGENIX's phylogenomic inference approach, three phylogenetic trees are produced, with three different approaches, the Neighbor Joining (NJ) method [31], the Maximum Parsimony (MP) method [32], and the "Quartet Puzzling" Maximum Likelihood (ML) method [33]. Usually, at the end of this step, an expert biologist manually examines the topology of each tree, runs different tests to compare trees one to one and finally tries to produce a projection onto a unique consensus topology of all the information from the three trees. This process is necessary to check whether the three reconstruction methods give congruent results or only partially congruent subtrees of the original trees. Depending on these congruence tests, conclusion could be drawn for the whole tree or only for subtrees. It also allows evaluating the reliability of the tree.

In the phylogenomic inference pipeline, two EK units give to the expert system the results of NJ, MP and ML topologies comparison tests produced by the automatically launched "TEMPLETON winning sites" test [20] and KISHINO-HASEGAWA test [34]. One data is given by test and by tree: (a numerical value or the label "best" showing which of the three trees has the "best" topology). Thus we get 6 data, from which an ED unit asks to expert system which fusion must be done in a consensus tree (Table 2).

**Table 2:** Data allowing export system to decide what kind of fusion must be done

***Kishino-Hasegawa***	***Templeton***
*Neighbor joining ****0.1266***	*Neighbor joining ****0.2170***
*Maximum Parsimony ****best***	*Maximum Parsimony ****best***
*Maximum likelihood *<0.0001*	*Maximum likelihood ****0.0010***

In this conceptual example, Templeton and Kishino-Hasegawa tests' results indicate that the Maximum Parsimony phylogenetic tree's topology is the "best" one and only the Neighbor Joining topology is congruent with this one.

The knowledge to be modeled and the different possible cases are shown in Table 3 and Table 4, and the corresponding PROLOG code is shown and commented on Appendix 1.

**Table 3:** All possible cases provided by tree topologies comparison tests

**Method**	**T1/K1**			**T2/K2**		**T3/K3**
**Parsimony (p)**	Best	>=0.05	>=0.05	Best	Best	Best

**Neighbor joining (n)**	>=0.05	Best	>=0.05	<0.05	>=0.05	<0.05
**Maximum Likelihood (l)**	>=0.05	>=0.05	Best	>=0.05	<0.05	<0.05

T indicates support from Templeton test. K indicates support from Kishino-HaseGawa test. Values returned by the tests that are >=0.05 indicate that the fusion is possible for the considered reconstruction method.

**Table 4:** Interpretation of phylogenetic trees topologies comparison tests

**Fusion Cases**	**T1**	**T2**	**T3**
**K1**	Case 1: 3 trees fusion on NJ labeled npl_A	Case 3: 3 trees fusion on NJ labeled npl_A	Case 5: 3 trees fusion on NJ labeled npl_K
**K2**	Case 2: 3 trees fusion on NJ labeled npl_A	Case 9: «best» tree if the same in the two tests with congruent tree labeled: pl or lp or nl or ln or np or pn	Case 6: no fusion
**K3**	Case 4: 3 trees fusion on NJ labeled npl_T	Case 7: no fusion	Case 8: no fusion

"T" indicates support from Templeton test. "K" indicates support from Kishino-HaseGawa test. "A" means support from all the tests. Suffix "1" indicates full congruence, "2" partial congruence, "3" non congruence.

#### Example 2: multiple alignment masking for sites not evolving under neutrality

At step 2 of phylogenomic inference approaches, a multiple alignment of putative homologous sequences is produced. Before being sent for phylogenetic reconstruction, multiple alignments need to be corrected for various different biases. Among those corrections, sites having high rates of evolution must be removed from the multiple alignment. Similarly, sites for which the rate of substitution is highly divergent in two or more paralogous groups, underlying a possible "non neutrality", should also be removed. Indeed phylogenetic reconstruction methods are not tolerant to sites highly divergent to neutral evolution and molecular clock. Sites not respecting this rule potentially produce errors in trees' reconstruction; they thus have to be masked.

**Figure 4:**
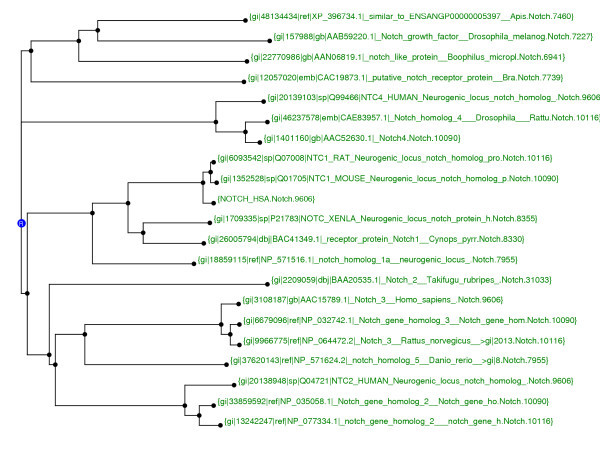
**Intermediate domain tree (NJ). **This tree, built with Neighbor Joining method, is used by expert module to detect paralogy groups. The reconstruction was made with Human Notch1 as query on the NCBI NR database. Here we have three significant groups tagged "G" on the figure (species taxon end the labels).

In the FIGENIX's phylogenomic inference pipeline, we use the "Functional divergence test" [35] (at individual domain level) to detect sites not evolving under neutrality. An EK unit gives to the expert system a phylogenetic tree (see figure 4), built with Neighbor Joining algorithm from the dataset alignment area associated with a specific domain. An ED unit asks the expert system to determine in this tree the paralogy groups. Once these paralogy groups are determined, divergence test [35] is applied to all of them to get the sites (site <=> amino acid column in the multiple alignment) that don't respect the rule of evolution under neutrality.

Biologists use to determine theses groups by just looking at the tree. After doing an in depth analysis of their experiment and reasoning, it seems that the knowledge to be modeled can be summarized in this sentence: "Paralogy groups contained in a phylogenetic tree are the biggest sub-trees containing sequences from different species (sequences groups containing only one species are equivalent to a unique node), but containing no sequence belonging to the species chosen as "out group" parameter by the biologist if any"

This is typically the kind of knowledge that can be modeled in the expert system and that is detailed in Appendix 2.

These two examples, clearly show the interest of this approach for knowledge and reasoning modeling in a very few and easily maintainable concise ruleset. These examples are taken from the phylogenomic inference pipeline which is intentionally over-summarized in this section into 5 main steps (detailed on the supplement). The whole phylogenomic inference pipeline included in FIGENIX contains 50 different steps (figure 2). Each of these steps automates processes usually requiring manual intervention of a biologist, 14 of these steps represented by "expert steps" require expert biologists' knowledge and decisions. This last category of steps accounted to date for the main difficulties in automating pipelines such as the one described here in their whole complexity.

## Results and discussion

### Results

FIGENIX currently proposes 8 pipeline models allowing both structural and functional annotation. While the architecture and design of the platform do not restrict its usage for a particular scope of species, the pipelines currently available are more suitable for eukaryotic species, and this is especially the case for structural annotation pipelines. This is due to the fact that research in our laboratory is more eukaryotes-centered, but specific pipelines designed by experts of prokaryotic genomics could easily be integrated in the flexible architecture of FIGENIX. A complete list of the pipelines available today is presented on Table 5. The type of data that can be used as an entry to the various different pipelines is, depending on the pipeline used, virtually any FASTA sequence (or set of sequences), ranging from ESTs to cosmids, scaffolds or genomic region for nucleotides, or any number of protein sequences from any species for amino-acids based pipelines. The only limitation, as discussed later is the size of the input sequence which depends on the available computational power.

**Table 5:** The 8 pipeline models currently available in FIGENIX

Pipeline Name	Pipeline Purpose
ProtPhyloGenix	The phylogenomic functional inference pipeline shown in this paper and detailed in the supplement.
TwinBaseMatix	Builds a FASTA database, eliminating redundant sequences obtained from two different query databases. For example, mixes protein coming from NR and Ensembl databases, and eliminates doubles.
BaseProtPhylogenix	Composition of the two previous pipelines. This pipeline first builds a temporary protein database (mixing two different databases and eliminating doubles). The phylogenomic inference process is then run using the built database.
TwinESTMatix	Builds a FASTA database, mixing sequences obtained on the one hand from a filtered given database and on the other hand by a database of automatically clustered ESTs. For example, it allows mixing protein coming from NR and translations of EST contigs from NCBI dbEST database.
BaseESTPhylogenix	Composition of TwinESTMatix and ProtPhyloGenix__ pipelines. Phylogenomic inference on FASTA databases built with TwinESTMatix This allows construction of phylogenetic tress mixing proteins and translated EST contigs.
GenePredix	Runs our structural annotation method (mixing ab-initio and homology information) to DNA sequence up to ~50 kb (due to current computational power limitations) to predict genes. For larger DNA sequences, SlidingGenePredix can be used.
SlidingGenePredix:	Apply the GenePredix pipeline on a sliding window. This allows gene prediction on larger DNA sequences, and bypasses the ~50 kb limitation.
PhyloGenix:	Composition of GenePredix and ProtPhyloGenix pipelines. This model allows automatic structural and functional annotation of DNA sequences. Indeed it produces gene prediction in DNA sequences using GenePredix, and then performs phylogenomic functional inference for each putative gene using ProtPhyloGenix.

### Validation and performance of FIGENIX's results

Complete automation of complex pipelines through the use of an expert system, although providing obvious gains in time cost, does not necessarily presume of the quality of the produced results. We addressed this question by evaluating the quality of the results and the performance of FIGENIX's pipelines.

### Structural annotation results

With regards to the structural annotation pipeline, FIGENIX has already been used to produce results published in peer-reviewed journals. For example we annotated several amphioxus cosmids [36] and Ciona savignyi scaffolds [37] from which we deciphered several genes whose orthologs are found in human in the Major Histocompatibility Complex (MHC) or paralogous regions. In parallel we also evaluated specificity and sensitivity of our method in comparison to two widely used ab-initio methods, Genscan [17] and Hmmgene [18] (Table 6). Results are of course more specific and sensitive than ab-initio methods used alone since our hybrid approach includes homology based predictions. Our approach resembles the one used in Procrustes [38], with two main differences:

**Table 6:** Performance of two Ab-initio methods vs. FIGENIX's structural annotation method

**Program**	**Initial exons (55)**	**Internal exons (186)**	**Terminal exons (55)**	**False positive (overprediction)**	**Correct full length protein prediction**
**Genscan**	0.55	0.80	0.65	0.22	0.31
**HMMGene**	0.75	0.81	0.78	0.15	0.38
**FIGENIX**	0.91	0.92	0.95	0.05	0.87

Performances were measured on a modified version of the HMR195 [53] dataset. The new dataset contains 55 sequences from Mouse and Rat genomes. They were annotated with Genscan, HMMGene and FIGENIX (with the human section of Swissprot [54] as a reference database for homology-based approach).

- The platform chooses itself from a BLASTX output the reference protein sequence to compare to raw DNA sequence for gene prediction.

- Extension of BLAST's high scoring pairs (HSPs) to splice donor and acceptor sites, start and stop codon is done under supervision of the expert system, as well as the alignment of predicted proteins with the reference protein.

### Phylogenomic inference results

Concerning the phylogenomic inference pipeline, several phylogenies produced by FIGENIX have already been validated in peer reviewed article [39]. The results of these phylogenies turned out to be congruent with previously published phylogenies (e.g. the PSME, TAP and GRP78 families [40,41]). Additionally, as the pipeline automated and implemented in the platform is based on the methods developed in our lab and published in 2002 [29]; we compared the phylogenies produced today by FIGENIX's pipeline to the 31 trees published in 2002 [29] and to the 38 in 2003 [30] that were all manually produced in our lab. All the trees produced by FIGENIX led to the same orthologs and paralogs inference than the 69 trees published in 2002 and 2003, with similar confidence (bootstrap) values, and with obviously additional sequences in the phylogenies produced today due to automatically updated databases in FIGENIX. In this case also, phylogenies produced by the platform where congruent (with additional species) with previously published phylogenies (e.g. the RXR, Notch, C3-4-5, PBX, and LMP families [41]). The quality of the phylogenies produced by FIGENIX's pipeline can thus be compared to the one of phylogenies produced by expert biologists through the manual chaining of algorithmic tools and software. The major difference is that, while it usually takes one to several weeks to manually produce phylogenies of this quality, it takes minutes to few hours with FIGENIX.

**Figure 5:**
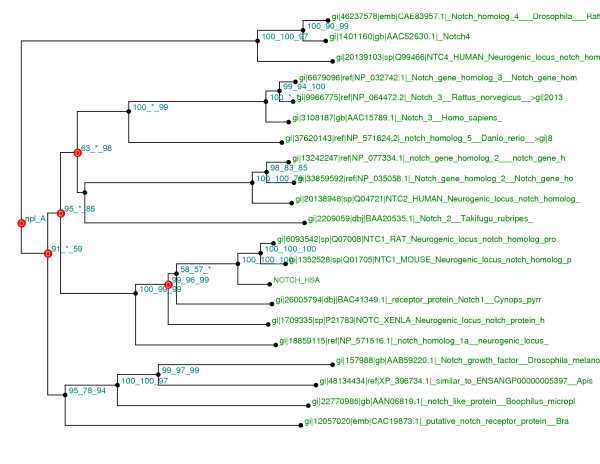
**Consensus phylogenetic tree of the Notch family. **The tree is midpoint rooted. At the root of the trees, a "npl_A" label means that the tree is the result of the fusion of three independently reconstructed trees with Neighbor Joining, Maximum Parsimony, and Maximum Likelihood methods. In this case, the fusion is done on the NJ topology (branches' lengths can be displayed but are not shown here to keep the tree easily readable). That means that topologies are strongly congruent. The bootstrap values are given for the three methods when a node exists as identical in the three trees. (sometimes a node exists only in two trees or only in the Neighbor Joining tree, e.g. a bootstrap 100_*_99 means that the node exists in NJ tree with a bootstrap value equal to 100 in ML tree with a bootstrap value equal to 99, but doesn't exist in the MP tree).

**Figure 6:**
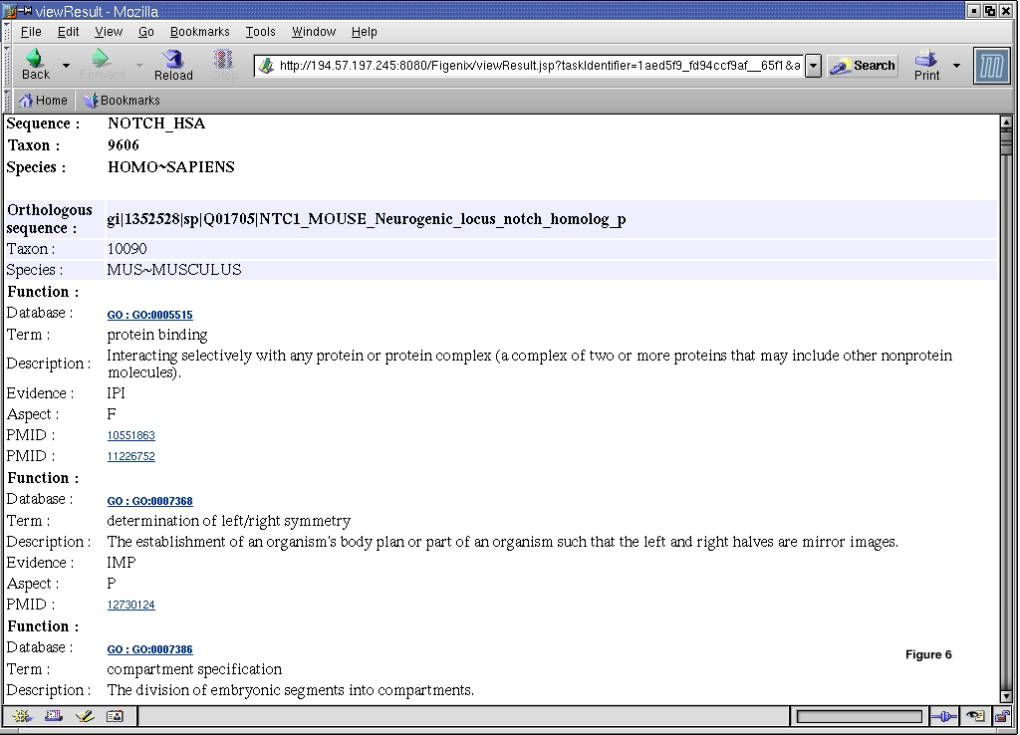
**Human Notch1 functional report. **The browser window shows a Web page with part of an automatically generated functional report. One of the orthologs (NTC1_MOUSE) to the query sequence (NOTCH_HSA) is shown, including some associated functional terms. At the end of each phylogenomic pipeline (Figure 5), after orthologs detection was produced on the consensus tree, an additional process is run. The goal of this process is to search on the Web experimentally verified functional data on proteins orthologous to the studied sequence. A HTML report synthesizing functional retrieved data is then built. It includes links to Web database and publication associated to retrieved functional terms. Current implementation of this system manages data coming from: GENE ONTOLOGY [51], MGD [52], and EST expression data available on NCBI Web site. This system is open for integration of other data sources.

We illustrate this gain in time cost with quality comparable to expert human-made phylogenetic analysis, with an example of phylogenetic reconstruction done on the Human Notch1 protein, with the phylogenomic inference pipeline (Figure 5) which is followed by an automatic research of known experimental data for orthologs to the query gene summarized in a "functional report" (Figure 6).

We don't show here all the intermediate results produced by the task's execution, nor details on parameters used for each tree building algorithm but FIGENIX users can consult, via the Web interface, all produced genomic results and associated parameters.

To automatically detect from the fusion-tree (figure 5) duplications (D-labeled nodes) and speciation (S-labeled nodes) events, we use the Forester (JAVA library) detection algorithm [42]. To compare our consensus tree with a reference tree, we don't use the tree of life given by the Forester library [42], but, instead, a minimum species tree dynamically extracted from a local copy of NCBI taxonomy's tree of life for each dataset (other reference trees can be chosen). Once duplications are detected, the platform automatically deduces sequences orthologous to the query sequence (here human Notch1 protein labeled "NOTCH_HSA"). At the end of this step, known and experimentally verified functions for all these sequences are automatically searched as shown in functional report on Figure 6.

The execution of the whole pipeline (run on the NR database) takes 25 minutes on the platform (running on a DELL POWEREDGE 1600SC dual-processor Xeon 2.4 Ghz with 1 GB Ram) The quality of the results can be compared to the one published in 2002 by Abi-Rached et al. on the notch family [29] that took around one week of processing by human expert biologists. The gain in terms of time cost here is evident and is obtained without compromising result quality.

### Input limitations

Performance and size limitations of the input sequence both depend on several parameters and on the type of pipeline used. For phylogenetic inference the size of the query protein, the number of homologs, and the number of domains all account in the global performance of the pipeline. Typically FIGENIX can handle phylogenomic inference tasks in less than an hour for protein up to 1000 amino acids and having up to 50 homologs. Concerning structural annotation pipelines, the size of the input sequence as well as the predicted gene density and complexity (in terms of number of exons/introns) all have an impact on the process's performance. To date, we have annotated amphioxus cosmids of sizes around 40 kb with a mean number of 5 predicted genes in less than half an hour per cosmid. We have already tested FIGENIX with several hundred kb long sequences [37], but not yet with longer genomic portions. The annotation of whole eukaryotic genomes would probably need more computational power. However, the global architecture of the platform has been designed to support multiple CPU and can thus potentially handle annotation of whole genomes with appropriate computational power.

### Pairwise-based vs. phylogenomic-based homology prediction methods

Methods like Inparanoid [9] or Homologene [13] already exist to automatically find orthologs and paralogs to a sequence of interest. From these methods, biologists could then extract known biological function of detected orthologous genes to infer, as we do, a biological function to the query sequence. However, as expected [2-5], these methods based on pairwise similarity suffer from limitations compared to multiple alignments and phylogeny based methods such as the phylogenomic inference pipeline currently included in FIGENIX. The major problem shared by these two approaches is that none give a representation of the evolutionary history and behavior of the genes. Thus, possibly useful information to understand gene function are missed, such as, for example, the detection of sites responsible of functional divergence between two groups of paralogy or the evaluation of the rate of evolution possibly indicating functional shifts between homologous genes. Another drawback of these approaches is that they are unable to correctly manage differential losses of paralogous and orthologous genes between different species [43,44]. These two approaches also have specific limitations. For example, Inparanoid only allows two by two comparisons of proteomes and requires that genomes compared are fully sequenced and annotated with high quality, which reduces the scope of usable data. However, when all these requirements are fulfilled, Inparanoid produces orthologs predictions with high specificity and sensibility [45], and is able to distinguish in-paralogs, out-paralogs, and orthologs. With regards to Homologene, the problem is different; it allows multiple species comparisons but the system is unable in a non-negligible proportion to provide all the orthologs that would be found through a phylogenetic reconstruction. What is more this system does not consider phylum-specific duplications and is unable to predict paralogy and co-orthology relationships. This problem is illustrated in Table 7 in which we compared orthologs found by Homologene to orthologs and paralogs found with our pipeline for the set of MHC-related genes we published in 2004 [39] and on the Notch family taken as an example in this paper. As shown on table 7, approaches like Homologene give rather reliable predictions of orthologs when single copy genes are studied. In contrast, such approaches systematically fail to detect specific genes duplications and are thus unable to find paralogous genes. For example, Homologene fails to detect human Notch2, Notch3 and Notch4 as co-orthologs to drosophila N gene. Homologene considers Notch1 as the only human ortholog to Drosophila N gene. In the case of multiple-copy genes, using such approaches to infer functional data for a gene can be misleading. Indeed after duplication, paralogous genes that are fixed in evolution usually undergo either neo-functionalization or sub-functionalization, compared to the original function of the ancestral gene [46].

**Table 7:** Specific differences between FIGENIX's phylogenomic inference pipeline and other software

**FIGENIX**	**RIO**	**PhyloGenie**
Homologous sequences search on any NCBI-formatted database including nr, Swissprot and Ensembl.	Homologous sequences search limited to Swissprot and trEMBL.	Homologous sequences search on any NCBI-formatted database including nr.
Choice of the scope of phylomes by the user (root = all phylomes by default)	No choice of the scope of phylomes by the user.	Choice of the scope of phylomes by the user.
Automatic detection of domains on the query sequence.	Manual input of a domain that must be present in pfam and for which pairwise distances must have been precalculated.	Phylogenetic reconstruction at BLAST's high scoring pairs (HSPs) level converted after corrections in multiple sequence alignment (MSA).
Expert system selection of domains and repeats whose evolutionary behaviour are congruent.	Phylogenetic reconstruction on a single domain provided by the user.	No test for domains congruence. Phylogenies constructed on a corrected alignment with a HMM profile.
When no domain is found phylogenetic reconstruction on the "alignable" portion of the query sequence.	No reconstruction possible when no known domain is present on the query sequence.	Phylogenetic reconstruction possible regardless the presence of a known domain on the query sequence.
Elimination of sites not evolving under neutral evolution.	No elimination of sites producing biases in phylogenetic reconstruction.	No elimination of sites producing biases in phylogenetic reconstruction.
Elimination of sequences having a divergent amino acids composition	No elimination of sequences with divergent composition.	No test for sequence composition but selection for sequences producing significant alignments with the query HMM.
Phylogenetic reconstruction with three different methods and projection on a consensus tree.	Phylogenetic reconstruction with one single method (NJ).	Choice of reconstruction method (NJ by default) but only one method at a time and no fusion with multiple methods.
Comparison of the consensus tree with NCBI reference tree of life containing around 200,000 taxa.	Comparison of the NJ tree with a reference tree of life containing around 2,500 taxa.	Comparison of the one-method tree with NCBI reference tree of life containing around 200,000 taxa.
Automatic detection of speciation and duplications, of orthologs and paralogs.	Automatic detection of speciation and duplications, of orthologs and paralogs.	Functionality not available. Possibility to scan a database of trees for a given topology.
Automatic extraction of experimentally verified functional information for all detected orthologs and paralogs.	Functionality not available	Functionality not available

While comparison between pairwise-based and phylogenomic-based approaches to detect homology relationship can appear biased, it illustrates what kind of information is missed by the widely-used pairwise approaches and what kind of systematic errors they are likely to produce and spread on biological databases. Comparison of FIGENIX's pipeline with other automated phylogenomic inference software is discussed in the next section.

## Discussion

In the field of structural and functional annotation, Ensembl [12] or BioPipe [47] automated systems propose quite similar frameworks, but independently of implementation's differences that were detailed previously, FIGENIX adds a new concept concretized by expertise units (or E units) which are responsible of crucial points in annotation process automation. They constitute "native" expert module gateways that do not have their counterpart in the Ensembl or BioPipe architectures. Such architectures thus still abundantly require human expertise and cannot fully automate processes such as phylogenomics inference.

### Comparison with other software proposing expertise integration

Counter to Ensembl [12] or BioPipe [47], the overall approach in FIGENIX can somewhat be compared to MAGPIE system [48,49] which also includes a kind of expert system. However, FIGENIX automated pipelines are data flow circulating, in a specific order, through computation tools. The expert system acts punctually to take decisions, extract or correct data. In contrast, in the MAGPIE system, computations are done independently on asynchronously incoming data and a PROLOG daemon produces logical deductions, verifying them on the "from data" computed results.

Other major differences in the concept and architecture of these two systems can be listed. For example, while MAGPIE was designed for local installation on a biologist's workstation, FIGENIX was designed as a server made accessible through the internet without the need of installing any additional software than a JAVA 2 browser plugin.

Differences which are not at the architecture or conceptual level reside in the type of biological applications which have been integrated in these two different systems. While MAGPIE automates processes mainly dedicated to structural annotation, FIGENIX additionally integrates Phylogenomic inference pipelines.

### Comparison with other automated phylogenomic inference software

Phylogenomic inference is, as stated in Background, a labor-intensive, complex and highly human-dependant process. These are the main reasons why other processes of functional and homology inference which are less complex and more straightforward (ie pairwise-based), have been considered for automation. But, as seen in the previous section, these automated processes ignore some of the functional information that could be deciphered through phylogenomic inference.

However, other groups have already proposed the complete automation of phylogenetic reconstruction pipelines like RIO [50] or like PhyloGenie [14] to address simpler methods'issues. To illustrate benefits from the use of an expert system we will discuss here the differences between the processes automated in these software and the phylogenomic inference pipeline included in FIGENIX (Table 8). Both methods of phylogenetic analysis automation [14,50] tackle most of the drawbacks linked to pairwise-similarity based approaches, in particular they allow multispecies comparison and are able to detect duplications and thus the existence of paralogs and co-orthologs. They thus propose notable improvements to similarity based approaches and allow high throughput phylogenetic analysis. The major difference with our phylogenomic inference pipeline resides in our expert system that allows automation of a more complex and refined process including more bias corrections and proposing building of a consensus tree which is an intelligent projection of three topologies built by three independent methods (NJ, ML, MP). Existing methods propose either only one reconstruction method (NJ for RIO) or choice between several methods but only one by task and no comparison between multiple methods (PhyloGenie). The gains in term of reliability are obvious with topologies supported by three independent methods compared to trees supported by a single method.

**Table 8:** Comparison of homology inference between FIGENIX's pipeline and Homologene

**Gene Family**	Query Gene*	**Paralogy relationship missed**	**Co-orthology relationship missed**	**Orthologs not detected in Taxa**	**Different orthology assignment**
Notch	Human Notch1	Notch2, Notch3, and Notch4 are not detected as paralogs of Notch1.	Notch2, Notch3, and Notch4 are not detected as co-orthologous to Drosophila N.	Amphibian Ray-finned fish Cephalochordata Arachnida	3 different C.elegans genes are detected for Hs Notch1, Notch2, and Notch3, suggesting that duplications giving rise to this family took place before the divergence between protostomes and deuterostomes, and that Notch2, and Notch3 were lost in Drosophila.
Calnexin/Calreticulin	Human Calnexin	Calmegin and Calreticulin are not detected as paralogs of Calnexin.	Calmegin is not detected as a Human co-ortholog to Drosophila CG9906 gene.	Amphibian Ray-finned fish	Calmegin is detected to be orthologous to another Drosophila gene than CG9906, suggesting Calmegin and Calnexin already existed as two duplicates before the divergence between protostomes and deuterostomes and Calmegin was secondary lost in C. elegans
ENPEP/TRHDE/LNPEP/ERAP/LRAP/ANPEP	Human TRHDE	ENPEP, LNPEP, ERAP, LRAP, and ANPEP are not detected as paralogous to TRHDE.	None	None	Each human gene of this family has been assigned a distinct ortholog in protostomes (e.g. Drosophila) suggesting this multigenic family emerged before the separation of Protostomes and Deuterostomes.
PSMB5/PSMB8	Human PSMB5	PSMB8 is not detected as paralogous to PSMB5	PSMB8 is not detected as co-orthologous to the same Drosophila gene than PSMB5.	Ray-finned fish Avian Cephalochordata. Amphibian	PSMB5 and PSMB8 are each assigned a distinct Drosophila ortholog suggesting they already existed as two copies in the last common ancestor of human and Drosophila.
PSMB7/PSMB10	Human PSMB7	PSMB10 is not detected as paralogous to PSMB7.	PSMB10 is not detected as co-orthologous to the same Drosophila gene than PSMB7.	Ray-finned fish Avian Cephalochordata. Amphibian	PSMB7 and PSMB10 are each assigned a distinct Drosophila ortholog suggesting they already existed as two copies in the last common ancestor of human and Drosophila.
Cathepsins L, M, P, R	Human Cathepsin R	Cathepsins L, M and P are not detected as paralogous to Cathepsin R.	None	Amphibian Avian Ray-finned fish	Each cathepsin gene is assigned a distinct drosophila ortholog suggesting the cathepsin family emerged before the separation between human and Drosophila.
Tpp2	Human Tpp2	None (not a multigenic family)	None	Drosophila	None
ERP57 (GPR58)	Human GRP58	None (not a multigenic family)	None	Fungi Bovine Schistosoma Avian	None
HSPA5 (GRP78)	Human HSPA5	None (not a multigenic family)	None	Amphibian Aplysia Lepidopteran Avian Schistosoma	None
TAP1, TAP2, ABCB9, MDR1	Human TAP1	TAP2, ABCB9, and MDR1 are not detected as paralogous to TAP1.	None	Drosophila Avian Amphibian Ray-finned fish	TAP1, and TAP2 are each assigned a distinct C.elegans ortholog and none in Drosophila, suggesting there was already two copies of these genes in the last common ancestor of these two species, and that the two copies were secondary lost in the Drosophila lineage.
PSME1, PSME2, PSME3	Human PSME1	PSME2, and PSME3 are not detected as paralogous to PSME1.	None	Protostomes Ray-finned fish	None
THOP1, NLN	Human THOP1	NLN is not detected as paralogous to THOP1	NLN is not detected as co-orthologous to the same N.crassa gene than THOP1.	Amphibian Bacteria	None

*Query gene is identical to the Query gene we used for phylogenetic reconstruction with FIGENIX's phylogenomic inference pipeline.

FIGENIX's phylogenomic inference pipeline also has specific differences with each of the two methods (Table 8). None of the compared methods already available propose functionalities such as for example the fusion of trees constructed by different methods, tests on domains and repeats congruence and their evolutionary behavior.

## Conclusion

Reliable automation is an absolute necessity for structural and functional annotation of huge amounts of genomic data coming from increasingly prolific sequencing projects. Many automated pipelines or genomic annotation platforms already exist as an answer to various different biological questions. However, to the best of our knowledge, no publicly available pipeline or platform yet includes an expert system (with "artificial intelligence") allowing such complete automation or automation of more complex process as FIGENIX does. The FIGENIX platform has today the capacity of detecting protein coding genes in raw nucleic sequences, of inferring their putative function through phylogenomic inference, of clustering ESTs and integrating them in phylogenomic analysis as well as gathering associated expression data. Several other complex pipelines whose automation was impossible so far because of the absolute requirement of human intervention at several steps can now be considered through FIGENIX.

## Availability and requirements

• ***Project name:****FIGENIX*

• ***Project home page: ***

• ***Operating system(s):****Platform independent (accessible through a web browser)*

• ***Other requirements:****JAVA 1.4.2 JRE plugin for web browsers*.

• ***License:****free for academic users (contact us to request login and password), source code is available upon request under the GNU General Public License*.

• ***Any restrictions to use by non-academics:****collaboration contract needed*

## Authors' contributions

PG computationally translated all biological methods, protocols and concepts into automated pipelines in FIGENIX, he developed the expert system and the mutli-agents system he produced the vast majority of the code constituting the platform and drafted the manuscript. EGJD and PP co-supervised the whole development of the platform, they provided most of the biological concepts and methods included in the platform; they verified that biological procedures were correctly translated into computational pipelines; they decided the biological orientation of the platform as well as the pipelines to be integrated. PP drafted the manuscript. EGJD wrote most of the manuscript. VV participated in designing the structural annotation pipeline, she produced all the tests, checked the validity of gene predictions, and measured the performances of this pipeline, she wrote some parts of the code included in this pipeline. NB designed the EST-based multigenic families' annotation pipeline and she tested the validity of results. AG participated in designing parts of the phylogenomics annotation pipeline. All authors read and approved the final manuscript.

## Appendix

Appendix 1 – PROLOG code for the fusion of trees built with 3 different methods

We modeled biologists' interpretation in a very natural way in PROLOG by these rules:

*fusion(npl_A) :- full_congruence(templeton, _), full_congruence (kishino-hasegawa, _)*.

*fusion(npl_A) :- full_congruence (templeton, _), partial_congruence(kishino-hasegawa, _)*.

*fusion(npl_A) :- full_congruence (kishino-hasegawa, _), partial_congruence(templeton, _)*.

*fusion(npl_T) :- full_congruence (templeton, _), no_congruence(kishino-hasegawa, _)*.

*fusion(npl_K) :- full_congruence (kishino-hasegawa, _), no_congruence(templeton, _)*.

*fusion(no_fusion) :- partial_congruence(kishino-hasegawa, _), no_congruence(templeton, _)*.

*fusion(no_fusion) :- no_congruence(kishino-hasegawa, _), partial_congruence(templeton, _)*.

*fusion(no_fusion) :- no_congruence(kishino-hasegawa, _), no_congruence(templeton, _)*.

*fusion(Label) :- partial_congruence(kishino-hasegawa, Label), partial_congruence(templeton, Label)*.

*Val1 < 0.05*,

*Val2 >= 0.05*,

*concat_labels (Best, Label2, Label)*.

These rules can be easily maintained. For example, we can decide to do the fusion on the "best" tree and not always on NJ tree like we do today by default in the 5 first cases. Rules will so look like this:

fusion(FusionOnTheBestLabel) :-

*full_congruence(templeton, Best)*,

*full_congruence (kishino-hasegawa, Best)*,

*get_fusion_label(Best, FusionOnTheBestLabel)*.

Information brought by EK unit during the pipeline execution take a form like this:

*topology(NameOfTest, Best, [Label1, Val1], [Label2, Val2])*.

(e.g.: topology(templeton, n, [p, 0.15], [l, 0.01]). that means that for Templeton test, the tree with the best topology is the one built with Neighbor Joining, that tree built with Maximum Parsimony is congruent with a 0.15 rate and that the one built with Maximum Likelihood is congruent with a 0.01 rate.)

Here are the rules for congruence tests:

% congruence is full when comparing rates are higher or equal to the chosen threshold

full_congruence(Test, Best) :-

*topology(Test, Best, [_, Val1], [_, Val2])*,

*Val1 >= 0.05*,

*Val2 >= 0.05*.

% we have no congruence when comparing rates are lower than the chosen threshold

no_congruence(Test, Best) :-

*topology(Test, Best, [_, Val1], [_, Val2])*,

*Val1 < 0.05*,

*Val2 < 0.05*.

% congruence is partial when one of comparing rates is lower than the chosen threshold

% the label associated to the fusion type is just the concatenation of label for "best" (see before) tree and for its congruent tree

partial_congruence(Test, Label) :-

*topology(Test, Best, [Label1, Val1], [Label2, Val2])*,

*Val1 >= 0.05*,

*Val2 < 0.05*,

*concat_labels(Best, Label1, Label)*.

partial_congruence(Test, Label) :-

*topology(Test, Best, [Label1, Val1], [Label2, Val2])*,

Appendix 2 -Commented prolog code for paralogy groups' detection

Each node of domain's phylogenetic tree, given to the "expert system" by an EK- unit, can have many children but for implementation reasons, we code it as a binary tree. Each node is a term like this:

node(TheSpecies, LeftChild, RightChild)

In the annotated tree, each node knows how many sequences it contains and has the full list of the different species it includes:

node(NumberOfSequences, AllSpecies, LeftChild, RightChild)

The main PROLOG rule for groups' detection is:

% detecting paralogy groups in a phylogenetic tree implies annotating tree nodes with species information then searching biggest groups with different species

paralogy_groups(PhylogeneticTree, ParalogyGroups) :-

*subtree_species(PhylogeneticTree, AnnotedPhylogeneticTree)*,

*biggest_groups(AnnotedPhylogeneticTree, ParalogyGroups)*.

(Rules with the same signature express a "logical OR" between them)

% a leaf node which species is different as the one chosen as out group can belong to a paralogy group

% (*) ! character in a PROLOG rule means that if the first rule is successful, PROLOG engine doesn't try other rules with same signature

subtree_species(node(Species, no, no), noeud(1, [Species], no, no)) :-

*outgroup_species(OutgroupSpecies)*,

*Species ≠ OutgroupSpecies, !*.

% a leaf node which species is the same as the one chosen as out group can't belong to a paralogy group

*subtree_species(node(_, no, no), node(1, no, no, no)) :- !*.

% annotate a node which has only one child is equivalent to annotate this child

% (we have pseudo nodes to force binary structure)

*subtree_species(node(_, Child, no), AnnotatedNode) :- subtree_species(Child, AnnotatedNode), !*.

% annotate a sub-tree with two children is equivalent to annotate the children and to compile found species

subtree_species(node(Species, LeftChild, RightChild), node(N, SpeciesList, Left, Right)) :-

*subtree_species(LeftChild, Left)*,

*Left = node(NL, SpeciesListL, _, _)*,

*subtree_species(RightChild, Right)*,

*Right = node(NR, SpeciesListR, _, _)*,

compile_annotations(NL, SpeciesListL, NR, SpeciesListR, N, SpeciesList)

% two sub-trees with the same unique species merge in a leaf of this species

*compile_annotations(_, [Species], _, [Species], 1, [Species])*.

% if one of the two sub-trees is invalidated for merging, the compilation is a tree invalidated for merging

% however we compute the total number of sequences in the sub-tree

*compile_annotations(NL, no, NR, _, N, no)) :- is(N, NL + NR)*.

*compile_annotations(NL, _, NR, no, N, no)) :- is(N, NL + NR)*.

% *if no species is common between the two sub-trees, we can merge all species*

compile_annotations(NL, SpeciesListL, NR, SpeciesListR, N, SpeciesList)) :-

*is(N, NL + NR)*.

*intersection(SpeciesListL, SpeciesListR, CommonSpecies)*,

*CommonSpecies = []*,

*concat(SpeciesListL, SpeciesListR, SpeciesList)*.

% search biggest paralogy groups

biggest_groups(node(N, no, Child1, Child2), Groups) :-

*biggest_groups(Child1, Groups1)*,

*biggest_groups(Child2, Groups2)*,

*concat(Groups1, Groups2, Groups), !*.

% accept group if more than 4 different species

biggest_groups(Group, [Group]) :-

*Group = node(N, TaxeIds, _, _)*,

*diff(TaxeIds, no)*,

*N >= 4, !*.

% reject subtree as a group

biggest_groups(_, []).
